# Reawakening Retrocyclins: Ancestral Human Defensins Active Against HIV-1

**DOI:** 10.1371/journal.pbio.1000095

**Published:** 2009-04-28

**Authors:** Nitya Venkataraman, Amy L Cole, Piotr Ruchala, Alan J Waring, Robert I Lehrer, Olga Stuchlik, Jan Pohl, Alexander M Cole

**Affiliations:** 1 Department of Molecular Biology and Microbiology, Burnett College of Biomedical Sciences, University of Central Florida, Orlando, Florida, United States of America; 2 Department of Medicine, David Geffen School of Medicine, University of California Los Angeles, Los Angeles, California, United States of America; 3 Biotechnology Core Facility Branch, DSR, Centers for Disease Control and Prevention, Atlanta, Georgia, United States of America; Fred Hutchinson Cancer Research Center, United States of America

## Abstract

Human alpha and beta defensins contribute substantially to innate immune defenses against microbial and viral infections. Certain nonhuman primates also produce theta-defensins—18 residue cyclic peptides that act as HIV-1 entry inhibitors. Multiple human theta-defensin genes exist, but they harbor a premature termination codon that blocks translation. Consequently, the theta-defensins (retrocyclins) encoded within the human genome are not expressed as peptides. In vivo production of theta-defensins in rhesus macaques involves the post-translational ligation of two nonapeptides, each derived from a 12-residue “demidefensin” precursor. Neither the mechanism of this unique process nor its existence in human cells is known. To ascertain if human cells retained the ability to process demidefensins, we transfected human promyelocytic cells with plasmids containing repaired retrocyclin-like genes. The expected peptides were isolated, their sequences were verified by mass spectrometric analyses, and their anti-HIV-1 activity was confirmed in vitro. Our study reveals for the first time, to our knowledge, that human cells have the ability to make cyclic theta-defensins. Given this evidence that human cells could make theta-defensins, we attempted to restore endogenous expression of retrocyclin peptides. Since human theta-defensin genes are transcribed, we used aminoglycosides to read-through the premature termination codon found in the mRNA transcripts. This treatment induced the production of intact, bioactive retrocyclin-1 peptide by human epithelial cells and cervicovaginal tissues. The ability to reawaken retrocyclin genes from their 7 million years of slumber using aminoglycosides could provide a novel way to secure enhanced resistance to HIV-1 infection.

## Author Summary

Defensins are a large family of small antimicrobial peptides that contribute to host defense against a broad spectrum of pathogens. In primates, defensins are divided into three subfamilies—alpha, beta, and theta—on the basis of their disulfide bonding pattern. Theta-defensins were the most recently identified defensin subfamily, isolated initially from white blood cells and bone marrow of rhesus monkeys. They are the only known cyclic peptides in mammals and act primarily by preventing viruses such as HIV-1 from entering cells. Whereas theta-defensin genes are intact in Old World monkeys, in humans they have a premature stop codon that prevents their expression; they thus exist as pseudogenes. In this work, we reveal that, upon correction of the premature termination codon in theta-defensin pseudogenes, human myeloid cells produce cyclic, antiviral peptides (which we have termed “retrocyclins”), indicating that the cells retain the intact machinery to make cyclic peptides. Furthermore, we exploited the ability of aminoglycoside antibiotics to read-through the premature termination codon within retrocyclin transcripts to produce functional peptides that are active against HIV-1. Given that the endogenous production of retrocyclins could also be restored in human cervicovaginal tissues, we propose that aminoglycoside-based topical microbicides might be useful in preventing sexual transmission of HIV-1.

## Abstract 3

Repairing an ancestral human pseudogene by mutagenesis, or by the application of aminoglycosides to suppress the termination codon, can restore the production of retrocyclins, which are peptides of the defensin family that are remarkable inhibitors of HIV-1 entry into cells.

## Introduction

Nearly 33 million people are infected with HIV worldwide [1,2], and despite extensive efforts there are no effective vaccines or other countermeasures to protect against HIV transmission [3]. In our attempts to find effective anti-HIV agents, our group determined that certain synthetic θ-defensins called “retrocyclins” are potent inhibitors of HIV-1 infection [4–8]. Retrocyclins belong to a large family of antimicrobial peptides known as defensins, all of which are cationic, tri-disulfide bonded peptides that have important roles in innate host defense. On the basis of the position of the cysteines and the disulfide bonding pattern, defensins are grouped into three subfamilies: α-defensins, β-defensins, and θ-defensins [9,10].

θ-Defensins such as retrocyclin have a cyclic peptide backbone, derived from the head-to-tail-ligation of two peptides that each contributes nine amino acids to form the 18-residue mature peptide [11]. θ-Defensins are the only known cyclic peptides in mammals and were originally isolated from rhesus macaque leukocytes and bone marrow [11–13]. While θ-defensin peptides are produced in old world monkeys and orangutans, in humans they exist only as expressed pseudogenes [14]. A premature termination codon in the signal peptide portion of human retrocyclin mRNA prevents its translation. The retrocyclin gene is otherwise remarkably intact, showing 89.4% identity with rhesus θ-defensins. Its genetic information was utilized to recreate retrocyclins synthetically and confirm their activity against both X4 and R5 strains of HIV-1 [4–7].

Retrocyclins inhibit the fusion of HIV-1 Env by selectively binding to the C-terminal heptad repeat region on gp41 blocking 6-helix bundle formation [15,16]. RC-101 is a congener of retrocyclin with a single arginine to lysine substitution that retains structural and functional similarity to retrocyclin [4]. RC-101 exhibited enhanced anti-HIV-1 activity against over two dozen primary isolates from several clades [7,8], and did not induce inflammation or toxicity in organotypic models of human cervicovaginal tissue [17]. Continuous passaging of HIV-1 BaL in the presence of subinhibitory concentrations of RC-101 for 100 days induced only minimal viral resistance [18]. Given these beneficial attributes, we envisioned that restoring the endogenous expression of retrocyclins in humans would provide an effective and natural way of combating HIV-1 infection.

In the current study we restored the translation of this evolutionarily lost retrocyclin peptide by ablating the premature termination codon using site-directed mutagenesis, and analyzed whether human cells can synthesize biologically active retrocyclins. We found that promyelocytic HL60 cells stably transfected with retrocyclin constructs in which the premature termination codon was corrected could express retrocyclins. Application of the expressed retrocyclins to TZM-bl cells, PM1 cells, and peripheral blood mononuclear cells (PBMCs) conferred protection against HIV-1 infection. Moreover, mass spectrometric techniques confirmed the presence of correctly folded mature retrocyclin peptides. We also explored methods to read-through the premature termination codon within the retrocyclin pseudogene. Previous reports revealed that aminoglycoside antibiotics could suppress the termination codon of pseudogenes and disease-associated nonsense mutations [19–25]. In bacteria, aminoglycosides bind strongly to the decoding site on the 16S rRNA, thereby hindering protein synthesis [26]. However, in eukaryotes, aminoglycosides bind to the eukaryotic decoding site with low affinity and induce a low level of translational misreading, which suppresses the termination codon through the incorporation of an amino acid in its place [27]. Herein, we utilized aminoglycosides to induce translational read-through of the θ-defensin pseudogene, which restored the expression of functional anti-HIV-1 retrocyclin peptides in human cervicovaginal tissue models. Topical application of aminoglycosides to produce endogenous retrocyclins in the vaginal mucosa might soon be an effective preventative to combat sexual transmission of HIV-1.

## Results and Discussion

### Creation of Promyelocytic Cells Stably Transfected with Retrocyclin Constructs

θ-Defensins are formed by post-translational modification of two 12-residue gene products, each of which is processed to give a nonapeptide that contains three cysteines. The N-terminus of one nonapeptide forms a peptide bond with the C-terminus of another nonapeptide, resulting in a cyclic 18 residue peptide with three intramolecular disulfide bonds [11,14]. To determine if human cells have retained the ability to process θ-defensins, we transfected promyelocytic HL60 cells with retrocyclin constructs each encoding a nonapeptide in which the premature termination codon was replaced with a glutamine (⊗17Q).

**Figure 1:**
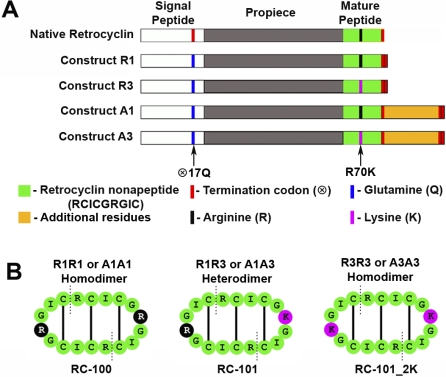
Design of Retrocyclin Constructs (A) Shows a schematic of the four constructs (R1, R3, A1, and A3) used for stable transfections along with native retrocyclin cDNA. All constructs have two termination codons at the end to ensure read-fidelity (red). Constructs A1 and A3 contain additional downstream residues (orange), whereas constructs R1 and R3 lack them. The two arrows indicate the position at which the two site-directed mutagenesis (⊗17Q and R70K) were performed. (B) Shows the three possible mature retrocyclin peptides that could be formed from the constructs, homodimers of R1 or A1 encoding RC-100 (wild-type retrocyclin), heterodimers of A1 and A3 or R1 and R3 encoding RC-101 (single lysine congener), and homodimers of R3 or A3 encoding RC-101_2K (double lysine congener).

Four types of constructs were produced: R1, R3, A1, and A3 (Figure 1). Aside from the corrected premature termination codon (⊗17Q), all constructs were engineered to contain two termination codons at the end of the gene to ensure read-fidelity. Constructs with an “R” designation terminate after the retrocyclin portion of the gene, while constructs with an “A” designation contain the retrocyclin portion with additional downstream residues that might be critical for translation and/or processing [14,28]. Constructs with a “1” designation do not have any additional residues mutated, while constructs with a “3” designation have the additional Arg → Lys mutation (R70K) encoding the RC-101 nonapeptide. HL60 cells were cotransfected by electroporation with either R1 and R3, or A1 and A3, and propagated in the presence of G418 (300 μg/ml) to create stably transfected cell lines. Stable transfection was verified by analyzing genomic DNA and mRNA (Figure S1). Since two different constructs were cotransfected for each condition, combinatorially it would be possible to generate three different retrocyclin peptides as illustrated in Figure 1B. For example, if cells were cotransfected with the R1 and R3 constructs, they could theoretically generate a heterodimer (HL60 cells containing retrocyclin constructs R1 and R3 [R1R3]) or homodimers (R1R1 or R3R3).

### Extracts of Promyelocytic Cells Stably Transfected with Retrocyclin Constructs Are Active against HIV-1

**Figure 2:**
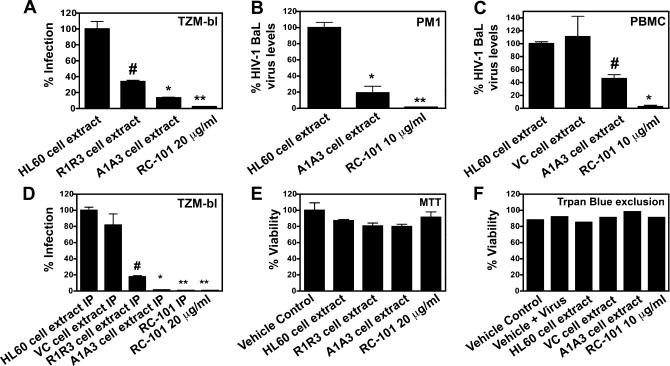
Extracts from HL60 Cells Stably Transfected with Retrocyclin Constructs Are Active against HIV-1 Infection (A) TZM-bl cells were treated with extracts or peptide as indicated in the figure and infected with HIV-1 BaL (6.5 ng/ml p24) for 24 h. Infection was measured as percent luciferase activity compared to cells treated with control cell extract (average relative luciferase units [RLUs] of control HL60 extract = 178,200). (B, C) PM1 cells and PBMCs were treated with extracts or peptide as indicated and infected with HIV-1 BaL (2 ng/ml p24) and cultured for 5–9 d. Bars represent percent BaL HIV-1 levels in the supernatants collected on days 5 (B) and 9 (C). The amount of p24 in PM1 cells treated with control extract = 76.85 ng/ml and in PBMCs treated with control extracts = 55.99 ng/ml. (D) TZM-bl cells were treated with immunopurified (IP) extracts or peptides as indicated and infected with BaL HIV-1 (p24 = 2 ng/ml) for 24 h. Infection was quantified as percent luciferase activity compared to cells treated with control HL60 cell IP extracts (average RLU = 764,460). Error bars represent standard error of the mean (SEM). *n* = 3–4; #, *p* < 0.004; *, *p* < 0.002; **, *p* <0.0005. (E) Cellular viability of TZM-bl cells treated with HL60 acid extracts as indicated was determined by measuring the reduction of MTT after 24h (*n* = 3). Bars represent percent viability as compared to vehicle control and error bars represent SEM. (F) Cell death was monitored in PBMCs treated with the acid extracts by a trypan blue exclusion assay on day 9 (*n* = 1).

We next analyzed if correcting the termination codon in the retrocyclin constructs could restore the translation of biologically active retrocyclin peptides. The infection of TZM-bl cells with HIV-1 BaL was significantly reduced when cells were treated with cellular acid extracts of R1R3 cells (*p <* 0.004) and HL60 cells containing retrocyclin constructs A1 and A3 (A1A3) (*p <* 0.002) (Figure 2A). A standard tetrazolium MTT assay revealed that the extracts did not affect cellular metabolism at the concentrations used in the experiment (Figure 2E). Addition of A1A3 cell extracts to HIV-1 infected PM1 cells (Figure 2B) and PBMCs (Figure 2C) showed significant (*p* < 0.002 and *p* < 0.004, respectively) decrease in the viral titer as compared to cells treated with control HL60 cell extract. A trypan blue exclusion assay was performed in PBMCs to monitor cell viability (Figure 2F). We next affinity purified R1R3 and A1A3 cell extracts using anti-RC-101 antibody and confirmed the antiviral activity in a luciferase-based assay system (Figure 2D). Interestingly, A1A3 cell extracts were found to be consistently more active than equivalent amounts of R1R3 cell extract, which suggests a role for the downstream residues in retrocyclin processing. These results indicate that biologically active recombinant retrocyclin peptides can be synthesized in human promyelocytic cells. As a next step we tested the presence of retrocyclin in promyelocytic cells using immunostaining.

### Immunofluorescence Staining of Stably Transfected HL60 Cells Reveals Retrocyclin Peptides

**Figure 3:**
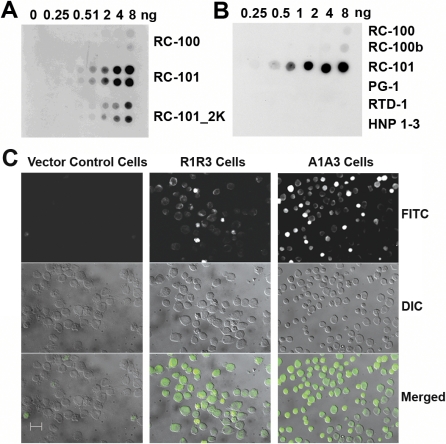
Immunofluorescence Staining of Stably Transfected HL60 Cells Reveals Retrocyclin Peptides (A) Retrocyclin peptides RC-100, RC-101, and RC-101_2K peptides (in duplicates) and (B) RC-100, RC-100b, RC-101, protegrin-1 (PG-1), rhesus theta defensin-1 (RTD-1), and human neutrophil peptides 1–3 (HNP 1–3) were dotted (0–8 ng/4 μl dot) on a PVDF membrane and subjected to immuno-dotblot analysis. (C) VC, R1R3, and A1A3 (100,000 cells each) were fixed onto glass slides and incubated with rabbit anti-RC-101 antibody followed by biotinylated goat anti-rabbit IgG secondary antibody and then stained using fluorescein isothiocyanate (FITC)-avidin. Slides were visualized using Zeiss Axiovert 200M microscope system at 40× magnification. The three rows show FITC staining, DIC, and the merged image, respectively. Scale bar represents 20 μm.

Immuno-dotblot analyses revealed that our anti-RC-101 antibody specifically recognized lysine-containing human retrocyclin analogs (synthetic RC-101 and RC-101_2K) and RC-100 (i.e., wild-type form) to a lesser extent (Figure 3A) but not human neutrophil peptides 1–3, or peptides with very similar tertiary structure including rhesus theta defensin-1 (RTD-1) and protegrin-1 (PG-1) (Figure 3B). This antibody was used to visualize the expressed retrocyclin peptides in the stably transfected HL60 cells by immunofluorescence staining, which revealed that R1R3 cells and A1A3 cells were brightly stained as compared to vector control (VC) cells (Figure 3C). Slides treated with preimmune serum showed no staining (unpublished data). Note that the staining of A1A3 was brighter than R1R3 and the morphology of A1A3 cells was smaller than VC cells. Experiments were next designed to purify and confirm the identity of the expressed retrocyclin peptides from the cell extracts.

### Stably Transfected Promyelocytic Cells Produce Retrocyclin Peptides

**Figure 4:**
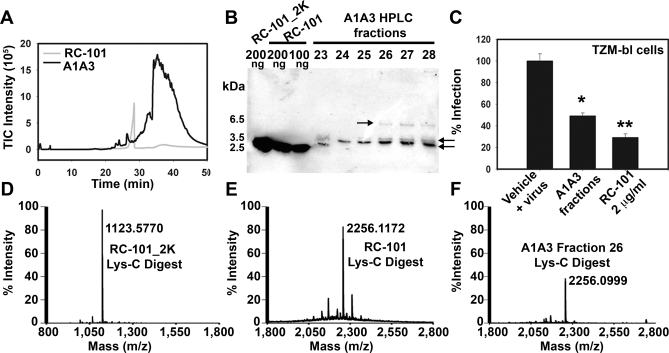
Stably Transfected Promyelocytic Cells Produce Retrocyclin (A) Shows the RP-HPLC trace of A1A3 cell extract (from 10^8^ cells) and 50 μg of synthetic RC-101. (B) Western blot of A1A3 HPLC fractions (23–28 min) using rabbit anti-RC-101 antibody. The arrows indicate the multimeric forms of retrocyclin observed in A1A3 fractions. (C) TZM-bl cells were infected with HIV-1 (p24 = 2 ng/ml) in the presence or absence of pooled A1A3 fractions (final dilution 1:6 in D10) or 2 μg/ml of RC-101 for 24 h. Infection was quantified by luciferase measurement (average RLU of vehicle control with virus = 85,450). Error bar represents SEM and *n* = 3–6; *, *p <* 0.0015; **, *p <*0.0001. MALDI-TOF MS spectra of Lys-C digested (D) synthetic RC-101_2K, (E) synthetic RC-101, and (F) A1A3 HPLC fraction 26 reveal that A1A3 cells produce RC-101.

Reverse-phase high-performance liquid chromatography (RP-HPLC) was utilized to purify the recombinant retrocyclin peptides from stably transfected HL60 cell extracts. Figure 4A shows the RP-HPLC trace of A1A3 and synthetic RC-101. Synthetic RC-101 was recovered in fractions collected at 26–28 min. A1A3 HPLC Fractions collected from 23–30 min were analyzed on a 16% Tricine-SDS-gel. Control samples did not contain any protein bands at the expected size, whereas fractions from R1R3 cell extracts revealed protein bands of about 6-kDa size (unpublished data). Interestingly, A1A3 HPLC fractions revealed multiple protein bands, which we further analyzed by western blot (Figure 4B). The western blot analysis revealed bands at sizes corresponding to a monomer, dimer, and trimer of retrocyclin. Interestingly, the presence of multimeric forms of retrocyclin has been independently observed by Daly and colleagues [29]. Furthermore, the RP-HPLC purified A1A3 fractions inhibited entry of HIV-1 BaL in TZM-bl cells (Figure 4C). The IC_50_ of retrocyclin peptides expressed by A1A3 cells (2 μg/ml) was similar to that of synthetic RC-101 (1.25 μg/ml) [8].

To determine the identity of the retrocyclin peptide expressed by A1A3 cells, HPLC fraction 26 was analyzed by mass spectrometric analysis (MALDI-TOF-MS) at the Microchemical and Proteomics Facility, Emory University (Atlanta, Georgia, US). Analysis of A1A3 Fraction 26 revealed peaks with masses 1,889.775 Da (oxidized) and 1895.890 Da (reduced), which is nearly identical to the expected mass of synthetic cyclic RC-101 (1,889.85 Da and 1,895.96 Da, respectively; unpublished data) and is in agreement with reduction of the three disulfide bridges in the molecule. Furthermore, treatment with iodoacetamide yielded mass species of 2,238.081 Da for the A1A3 fraction 26 and 2,238.071 Da for RC-101 corresponding to the predicted 6-fold–alkylated form of RC-101 (expected mass = 2,238.097 Da). Comparison of spectrum of the Lys-C digest of reduced/alkylated synthetic RC-101_2K (peak at 1,123.577 Da; peptide cleaved at two Lys-Gly bonds; Figure 4D), synthetic RC-101 (peak at 2,256.097 Da; peptide cleaved at a single Lys-Gly bond; N-terminal sequence determined as: Gly-Ile-Cys-Arg-; Figure 4E), and A1A3 fraction 26 (peak at 2,256.010 Da) suggests that the A1A3 cells are expressing RC-101 (Figure 4F). These data confirmed that correctly folded mature retrocyclin peptides can be expressed by human cells. In the following experiments we explored alternative methods to express the peptide endogenously. Of particular interest was the effect of aminoglycosides in mediating varying degrees of termination codon read-through as previously described [19–25].

### Aminoglycosides Mediate Read-Through of Termination Codon of Retrocyclin Gene and Restore Anti-HIV-1 Activity

**Figure 5:**
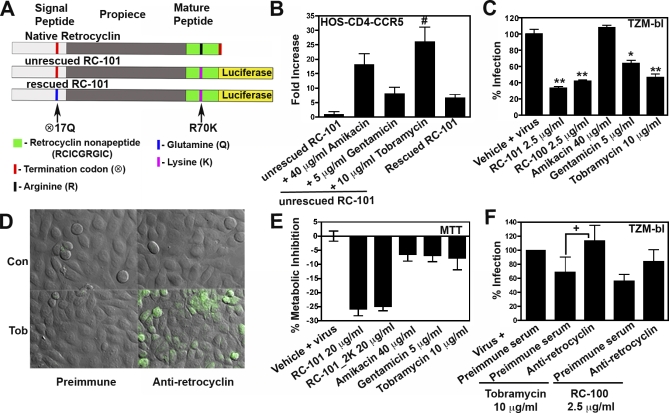
Aminoglycosides Mediate Read-Through of the Premature Termination Codon within the Retrocyclin Gene and Promote Anti-HIV-1 Activity (A) Shows a schematic of the luciferase fusion constructs unrescued RC-101 and rescued RC-101 along with native retrocyclin cDNA. (B) HOS-CD4-CCR5 cells cultured in antibiotic free medium (D10^−^) were transfected with unrescued RC-101 (negative control) or rescued RC-101 (positive control) plasmids along with phRL-CMV vector (transfection control). The next day transfected cells were treated with PBS for control cells or aminoglycosides at the indicated concentrations and allowed to grow for 24 h. Read-through was determined by measuring luciferase levels. Data are expressed as fold increase in luciferase expression normalized to renilla levels. (C) TZM-bl cells grown in D10^−^ were treated for 30 min with PBS, RC-101 (2.5 μg/ml), RC-100 (2.5 μg/ml), or aminoglycosides as shown in the figure and infected with HIV-1 BaL (2 ng/ml p24) for 24 h followed by luciferase measurement. Error bars represent SEM. *n* = 3–6; #, *p <* 0.007; * *p <* 0.0005; ** *p <*0.0001. (D) TZM-bl cells cultured on cover slips were treated with PBS (Con) or 10 μg/ml tobramycin (Tob) and then immunostained with rabbit preimmune or antiretrocyclin serum using a biotinylated secondary antibody FITC-avidin system. (E) Cellular cytotoxicity was assessed by performing an MTT assay on TZM-bl cells treated with indicated amount of peptide or aminoglycosides (*n* = 3). Bars represent percent metabolic inhibition as compared to control (vehicle + virus). (F) TZM-bl cells, treated with either PBS, tobramycin (10 μg/ml), or RC-100 (2.5 μg/ml), were incubated with preimmune serum or antiretrocyclin serum as indicated and infected with HIV-1 (p24 of 5 ng/ml). Data are represented as percent infection. Error bars represent SEM. *n* = 3; +, *p < 0.018* Statistical significance was determined by two-tailed Student's *t*-test.

We tested the ability of three commonly used aminoglycosides (gentamicin, amikacin, and tobramycin) to induce termination codon read-through of retrocyclin cDNA. The native retrocyclin gene was fused with a luciferase reporter at the C terminus to create two constructs: unrescued RC-101 and rescued RC-101 (positive control) as shown in Figure 5A. These constructs were transfected into HOS-CD4-CCR5 cells, grown in the presence of varying concentrations of aminoglycosides, and the degree of read-through quantified by measuring luciferase. Application of tobramycin (10 μg/ml) was the most effective, producing a 26-fold increase in read-through (*p* < 0.0007; Figure 5B).

Having thus established the optimal aminoglycoside concentration required to achieve read-through of retrocylin cDNA, we next determined if aminoglycosides could restore the translation and anti-HIV-1 activity of native retrocyclin peptides. HeLa-derived cells lines such as TZM-bl cells can natively express retrocyclin mRNA (unpublished data). We applied aminoglycosides to TZM-bl cells and challenged them with HIV-1 BaL. We found that cells treated with gentamicin and tobramycin significantly (*p* < 0.0005 and *p* < 0.0001, respectively) inhibited HIV-1 infection as compared to untreated cells (Figure 5C). The effect was modest when compared to inhibition by synthetic peptides. Cell viability, determined by a tetrazolium-based MTT assay, was not affected by the application of aminoglycosides at the mentioned concentrations (Figure 5E).

In order to visualize the retrocyclins expressed by application of aminoglycosides, we performed immunostaining. TZM-bl cells were treated with PBS control or 10 μg/ml tobramycin and stained with anti-retrocyclin antibody or preimmune serum. Control cells showed no staining while cells treated with tobramycin revealed brightly stained cells suggesting that aminoglycosides can induce the expression of retrocyclin peptides (Figure 5D).

We next incubated TZM-bl cells with tobramycin (10 μg/ml) for 24 h, and then treated the cells with preimmune or anti-retrocyclin serum followed by infection with HIV-1. Figure 5F reveals that cells treated with preimmune serum showed a modest yet significant reduction in infection as compared to cells treated with anti-retrocyclin antibodies (*p* < 0.018), suggesting that the antibody inhibited the endogenous retrocyclins. These data confirm that the anti-HIV-1 activity observed is due to the endogenous retrocyclin peptides expressed when tobramycin was applied to cells.

### Aminoglycosides Induce Production of Retrocyclin Peptides in Cervicovaginal Tissues

**Figure 6:**
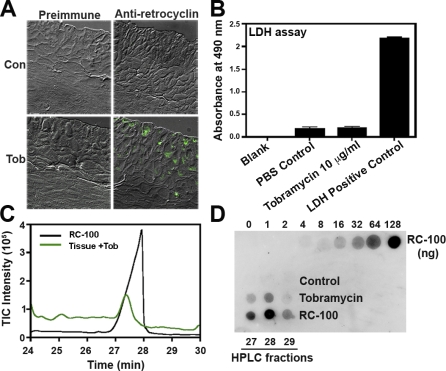
Expression of Retrocyclins in Cervicovaginal Tissue Model Using Aminoglycosides (A) Cervicovaginal tissues were treated with PBS (Con) or 10 μl tobramycin (Tob) and incubated with rabbit preimmune serum or antiretrocyclin antibody. The slides were then incubated with biotinylated goat anti-rabbit IgG secondary antibody and then stained using FITC-avidin. (B) Cytotoxicity was determined by measuring LDH activity in media underlying the tissues treated with PBS or tobramycin as indicated. Bars represent absorbance measured as 490 nm and error bars represent SEM; *n* = 6. (C) HPLC trace of extracts of tissues treated with 10 μg/ml tobramycin (tissue + Tob) and 20 μg of synthetic RC-100. (D) RC-100 synthetic peptide (indicated amounts), HPLC fractions 27–29 of control, tobramycin-treated, and RC-100 were dotted on a PVDF membrane and analyzed by immuno-dotblot.

We next analyzed the ability of aminoglycosides to induce the expression of retrocyclin peptides in an organotypic model cervicovaginal tissue. Tissues were treated apically with tobramycin or control (PBS) for 24 h and anti-retrocyclin immunohistochemical analysis was performed. Interestingly, tissues treated with tobramycin alone and stained with anti-retrocyclin antibody revealed brightly stained cells (Figure 6A) suggesting that production of retrocyclin peptides is induced upon application of aminoglycosides. Lactate dehydrogenase (LDH) activity in the medium underlying the tissues was performed to determine tissue cytotoxicity. The LDH assay revealed that application of 10 μg/ml tobramycin was not cytotoxic to the tissues (Figure 6B). In addition, treatment of tobramycin did not affect the metabolic activity adversely, which was determined by an MTT assay performed on one tissue (unpublished data).

In order to purify endogenous retrocyclins expressed in the tissues, we utilized RP-HPLC. Figure 6C shows an HPLC trace of control, tobramycin-treated tissue extracts as compared to synthetic RC-100 peptide. Synthetic RC-100 peptide was recovered in fractions collected at 27–29 min. Corresponding fractions from control and tobramycin-treated tissues were analyzed by immuno-dotblot analysis using the anti-RC-101 antibody. Figure 6D shows that retrocyclin peptides were recovered in fractions 27–29 min in tobramycin-treated tissue samples but not in control tissue samples. The amount of retrocyclin (RC-100) expressed in tobramycin-treated cervicovaginal tissues was estimated by densitometry to be approximately 1.6 μg/tissue. Together these studies show that aminoglycosides are promising molecules to suppress the premature termination codon of retrocyclin transcripts and restore the ability of cervicovaginal tissues to protect cells from HIV-1.

### Conclusion

Identifying effective drugs to prevent HIV-1 infection and other viral infections is essential for countering the spread of these diseases. Exogenous (synthetic) retrocyclins exhibit full activity in the complex environment of vaginal fluid and the peptide is very well tolerated in organotypic human cervicovaginal tissue models [17]. Moreover, HIV-1 evolves little resistance during continued passaging in the presence of the peptide [18]. For these and other reasons, retrocyclins have emerged as potential topical microbicides to protect against sexually transmitted HIV-1 infections.

In this study we have taken a different path towards developing θ-defensin therapeutics. The human pseudogenes that encode the demidefensin precursors whose post-translational processing gives rise to mature retrocyclin are expressed at the mRNA level in multiple organs, including the spleen, bone marrow, thymus, testis, and skeletal muscle [14], and cervicovaginal epithelia (A. M. Cole, unpublished data). By transfecting human myeloid cells with plasmids containing retrocyclin genes without a premature termination codon, we demonstrated that the “machinery” needed to process, trim, splice, and oxidize retrocyclin precursors was available in human myeloid cells. Two sets of expression constructs were transfected into cells: a shorter form (R1R3) that terminates at the end of the retrocyclin gene and a longer form that contains (A1A3) additional 3′ untranslated residues (UTR). Interestingly, A1A3 cells expressed higher levels of retrocyclin peptides as compared to R1R3 cells indicating a role for additional residues in the translational efficiency of these peptides. This was not altogether surprising as other studies have shown that the length of the 3′-UTR regulates translation efficiency [28,30]. Finally, we showed that aminoglycoside-treated cells and cervicovaginal tissues could produce retrocyclins endogenously by suppressing the premature termination codon in their endogenous mRNA transcript.

Since approximately 30% of inherited disorders may result from premature termination codon mutations, there has been tremendous interest and some progress in developing and applying agents that can read-through premature UAA, UAG, or UGA termination codons [25]. Although aminoglycosides, as used in this study, have been most widely investigated, exciting new agents such as PTC-124, have also appeared [31,32]. In a sense, human retrocyclin-deficiency is also an inherited disorder, albeit one with an incidence of 100%. It is caused by a premature termination codon mutation that occurred after human lineage diverged from the lineage we share with orangutans, lesser apes, and old world monkeys. Since HIV-1 and other viruses that currently infect humans have evolved in the absence of selective pressure exerted by retrocyclins, the ability to reawaken this ancestral molecule could be used to strengthen the innate immune system's ability to prevent or limit the infections they now induce.

## Materials and Methods

### Maintenance of cells, tissues, and viruses.

HL60 cells [33,34] obtained from ATCC were cultured in Iscoves's DMEM with 20% FBS, 100 U/ml penicillin, and 100 μg/ml streptomycin (I20). TZM-bl cells [35] stably expressing CD4, CCR5, and CXCR4, has firefly luciferase gene under the control of HIV-1 promoter (from J. C. Kappes, X. Wu, and Tranzyme Inc). TZM-bl, HOS-CD4-CCR5 [36,37] (from N. R. Landau), PM1 cells [38], (from M. Reitz), and HIV-1 BaL, an R5 tropic strain, were all procured through the National Institutes of Health (NIH) AIDS Research and Reference Reagent program. HIV-1 BaL viral stocks were prepared by infecting PM1 cells [18]. PBMCs were isolated from blood drawn from a healthy HIV-1 seronegative donor as per the guidelines of the institutional review board of University of Central Florida. PBMCs were isolated using Lymphosep (MP biomedicals LLC), and cultured in RPMI-1640 medium with 10% FBS (R10) supplemented with 50 units of IL-2 (R10-50U) and 5 μg/ml of phytohemagglutinin (PHA) for 3 d. The cells were then resuspended in R10-50U at a density of 0.8 × 10^6^ cells/ml and grown for 5–6 d.

Cervicovaginal tissues (EpiVaginal) were obtained from MatTek Corporation and maintained in proprietary growth medium as per the company's guidelines. The tissues were composed of a full-thickness, stratified vaginal-ectocervical layer intermixed with Langehans cells and underlying lamina propria. The tissues were allowed to grow on transwell cell culture inserts at the air-liquid interface.

#### Creation of retrocyclin constructs and stably transfected HL60 cells.

Retrocyclin cDNA was amplified from human bone marrow cDNA and cloned into pCRII-TOPO vector (Invitrogen). Two mutations, Termination codon (⊗17) → Gln (Q17) and Arg (R70) → Lys (K70) were introduced, either ⊗17Q alone (RC-100) or both (RC-101) using Quick change site-directed mutagenesis (Stratagene) and subcloned in-frame into the phCMV-luc-FSR vector (Genlantis) to generate four constructs R1, R3, A1, and A3 (Figure 1A). Plasmids R1 and A1 encode RC-100 nonapeptide while R3 and A3 encode RC-101 nonapeptide. Constructs A1 and A3 have a longer insert that includes additional downstream residues. HL60 cells (10^7^ cells/400 μl Iscove's DMEM) were cotransfected with 2 μg each of linearized R1, R3 or A1, A3, or phCMV-luc vector alone, by electroporation (exponential decay wave mode- 280 V; 975 μF) and selected in I20 medium with 300 μg/ml G418 sulfate. Stable transfectants thus produced were named according to the constructs used for cotransfection (R1R3, A1A3, or VC). Presence of these constructs in the cells was verified using PCR of genomic DNA (Figure S1A). PCR conditions used were the following: initial denaturation at 95 °C for 3 min; 30 cycles of 95 °C for 1 min; 58 °C for 1 min; 72 °C for 2 min; followed by a final extension at 72 °C for 7 min. Sequences of the primers used for the PCR reaction are listed in Table S1. RNA was extracted from 10^6^ cells (HL60, VC, R1R3, and A1A3) using TRIzol (Invitrogen), cleaned with DNaseI (Ambion Inc.), and cDNA synthesized (iScript, BioRad). Expression of recombinant genes was verified by PCR from the cDNA and subsequent restriction digestion using HpyCH4V (New England Biolabs) (Figure S1B and S1C).

#### Acid extraction and affinity purification of retrocyclin peptides.

HL60 cells (control, VC, R1R3, and A1A3) were extracted with 5% acetic acid by vortexing for 20 min, centrifuged for 10 min at 10,000 *g*, supernatants were then vacuum-dried and resuspended in 0.01% acetic acid. HL60 acid extracts (equivalent of 20 × 10^6^ cells) were affinity purified using anti-RC-101 polyclonal antisera immobilized to a Carbolink coupling gel (Pierce Biotechnology Inc.) prepared according to the manufacturer's instructions. Immunopurified samples were desalted using Sep-Pak C-18 cartridges (Waters). The elutes were then dried and resuspended in 100 μl of 0.01% acetic acid. 100 μg of synthetic RC-101 peptide was also affinity purified as positive control (RC-101 IP).

#### Luciferase-based infection assay to determine anti-HIV-1 activity.

TZM-bl cells (4,000 cells/well; 96-well plate) were infected with HIV-1 BaL (2–6.5 ng/ml of p24^gag^) in the presence of vehicle (0.01% acetic acid), HL60 extracts (from 0.25 × 10^6^ control, A1A3, or R1R3 cells), affinity purified extracts (from 0.625 × 10^6^ control HL60, VC, R1R3, A1A3 cells, or RC-101 IP diluted 1:32 times), or RC-101 (20 μg/ml) (positive control) for 24 h. Treatments were then removed and the infection was quantified by measuring luciferase using Bright-Glo reagents (Promega) in an LMax luminometer (Molecular Devices). Cytotoxicity and metabolic activity of cells were verified by a tetrazolium-based MTT assay (R&D Systems) performed on identically treated cells.

#### Antiviral infection assay in suspension cells and HIV-1 p24^gag^ ELISA.

Acid extracts of stably transfected HL60 cells were vacuum-dried and resuspended in PBS. PM1 cells (10^5^ cells) or PBMCs (10^6^ cells) were treated with PBS (vehicle) or HL60 extracts (from 10^4^ cells for PM1 and 10^5^ for PBMCs) of control cells or A1A3 cells or 10 μg/ml of synthetic RC-101 and infected with HIV-1 BaL (2 ng of p24/ml) in 100 μl of RPMI medium with 20% FBS (R20) for 2 h. Cells were then washed with 2 ml of R20, resuspended in fresh medium containing the treatments, and cultured for 5–9 d. Subsequently on alternate days culture supernatants were collected and fresh medium with the corresponding treatments was added. Viability of the cells was measured using trypan blue exclusion assay. Amount of HIV-1 virus in the culture supernatants was quantified by ELISA for HIV-1 p24^gag^ (Perkin Elmer).

#### Immuno-dotblot assay.

Peptides RC-100, RC-100b, RC-101, RC-101_2K, synthetic protegrin-1 (PG-1), Rhesus theta defensin–1 (RTD-1), and human neutrophil peptides 1–3 (HNP 1–3), or unknown samples were dotted (4 μl dot) as indicated on a 0.22-μm polyvinylidene fluoride (PVDF) membrane (Immobilon-P) that was activated with methanol and presoaked in TBS. The membrane was then probed with 1:1,000 rabbit anti-RC-101 antibody and developed using Immun-star HRP reagent (BioRad) [17].

#### Immunostaining of stably transfected HL60 cells using anti-RC-101 antibody.

HL60 cells (VC, R1R3, and A1A3) were fixed on slides (100,000 cells/slide), immersed in 10% Formalin in PBS for 10 min, washed (PBS for 2 min), incubated in Target retrieval solution (Dako North America Inc.) for 20 min at 95 °C, cooled to 25 °C, washed, blocked (2% Goat Serum, 0.1% Triton-X, 0.05% Tween-20, antibody buffer [10 mg/ml BSA/1 mg/ml gelatin/PBS]) for 20 min, and incubated in rabbit preimmune serum or rabbit anti-RC-101 antibody (1:5,000 in antibody buffer) overnight. Slides were washed, incubated in biotinylated goat anti-rabbit IgG antibody (1:20,000 in 1% goat serum/PBS for 30 min), followed by additional washing and treatment with Fluorescein-Avidin D (Vector Laboratories Inc.; 1:500 in PBS for 30 min). Cover slips were mounted using Vectashield fluorescence mounting medium and visualized using a Zeiss Axiovert 200M microscope system.

Tissues for immunofluorescence staining were fixed in 4% paraformaldehyde and slides were prepared by Mass Histology. The slides were deparaffinized, washed with TBS, and stained with anti-retrocyclin or preimmune serum and immunostained the same way as cells. The slides were then visualized on a Zeiss Axiovert 200M microscope system with 450 ms exposure time for all slides.

#### Separation of proteins from stably transfected HL60 extracts using RP-HPLC.

Acid extracts from control HL60 and A1A3 cells (equivalent of 100 × 10^6^ cells) were separated by RP-HPLC using the Alliance HT Waters 2795 Separations Module on a C_18_ Column equilibrated in solvent A (aqueous 0.1% TFA). Elution was done with a gradient of 0%–95% solvent B (0.08% TFA in acetonitrile), for 75 min, at 1 ml/min. Collected fractions (1 ml each) were vacuum dried and reconstituted in 100 μl of 0.01% acetic acid. Synthetic RC-101 peptide (control) was recovered from the fractions eluting at 26–28 min. A1A3 HPLC fractions (numbers 23–28) were electrophoresed on a 16% Tricine-SDS gel and electroblotted on a 0.22 μm PVDF membrane at 180 mÅ for 22 min. The western blot membrane was then processed as described [17] and developed with ChemiGlow reagent (Alpha Innotech). A1A3 RP-HPLC fractions (27–30 min) were pooled and the concentration was determined to be (2.13 ng/μl) by densitometry measurements using Quantity one 1-D analysis (BioRad). A luciferase-based assay was used to verify the activity of A1A3 HPLC fractions (diluted three times in D10) against HIV-1 BaL (2 ng p24/ml).

MatTek cervicovaginal tissues treated with PBS (control) or 10 μg/ml tobramycin were extracted with T-PER reagent (Pierce Biotechnology Inc.) and separated by RP-HPLC. 20 μg of synthetic retrocyclin (RC-100) was also separated as a positive control. Synthetic RC-100 was eluted in fractions collected at 27–29 min. Tissue samples eluted at 27–29 min were vacuum-dried to near dryness and resuspended in 100 μl of 0.01% acetic acid. HPLC fractions (27–29 min) of MatTek tissue extracts (control or tobramycin-treated) and synthetic RC-100 were analyzed by immuno-dotblot analysis.

#### Mass spectrometric analysis.

A1A3 HPLC Fraction 26, RC-101, and RC-101_2K were reduced, alkylated, and treated with Lys-C protease for 30 min before analyzing by mass spectrometry. In brief, 20 mM Tris [2-carboxyethyl] phosphine (TCEP) was used to reduce (30 min at 25 °C) the samples, alkylated by incubating the samples with iodoacetamide (60 mM; 45 min at 25 °C [pH 8–9]) followed by digestion with Lys-C (Wako Chemicals; 30 min at 37 °C) and subjected to MALDI-TOF-MS analysis using a model 4700 Proteomics Analyzer (Applied Biosystems) as described previously [39]. Lys-C digested RC-101 was desalted using C18 ZipTip (Millipore Corp.) and subjected to Edman degradation on cLC-Procise sequencer (Applied Biosystems).

#### Aminoglycoside mediated read-through of termination codon.

Wild-type and mutant retrocyclin cDNAs were subcloned into phCMV-luc-FSR vector to create unrescued RC-101 and rescued RC-101 C-terminal luciferase fusion constructs, and verified by sequencing. HOS-CD4-CCR5 cells were cultured in antibiotic free growth medium (D10^−^) and cotransfected with 0.5 μg of unrescued or rescued (positive control) RC-101 plasmids along with 0.1 μg of phRL-CMV vector (transfection control containing renilla luciferase gene) using Effectene transfection reagent (Qiagen). The next day cells were treated for 24 h with the appropriate aminoglycoside (40 μg/ml amikacin or 5 μg/ml gentamicin or 10 μg/ml tobramycin) or D10^−^ for control cells. Read-through was determined by measuring luciferase and renilla levels using a dual luciferase assay (Promega).

TZM-bl cells (4,000 cells/well; 96-well plate) were cultured in D10^−^ and treated with vehicle (PBS buffered D10^−^) or peptides RC-101 or RC-100 (2.5 μg/ml each) as positive control or aminoglycosides as before for 30 min followed by infection with HIV-1 BaL (p24 of 2 ng/ml) at 37 °C for 24 h. Subsequently, viral infection was quantified by measuring luciferase levels using Bright-Glo reagents (Promega). Cellular metabolism was monitored by measuring reduction the ability of cellular dehydrogenases to reduce MTT to formazan (R&D Systems).

TZM-bl cells were cultured on coverslips and treated with PBS control or 10 μg/ml of tobramycin for 24 h. The coverslips were then processed for immunofluorescence staining with anti-retrocyclin (rabbit anti-RC-101 antibody) or preimmune serum as described above.

For antibody-mediated neutralization experiments, TZM-bl cells (4,000 cells/well; 96-well plate) were cultured in D10^−^ medium and treated with vehicle (PBS) or 10 μg/ml of tobramycin for 24 h. The next day, cells were treated with either rabbit preimmune or anti-retrocyclin serum diluted 1:10 in D10^−^ medium containing tobramycin or RC-100 (2.5 μg/ml). 2 h later the cells were infected with HIV-1 BaL (p24 of 5 ng/ml) at 37 °C for 24 h. Viral infection was quantified as described above. An MTT assay was performed to confirm that the treatments were not cytotoxic (unpublished data).

#### Application of aminoglycosides to organotypic cervicovaginal tissue model.

Cervicovaginal tissues were treated topically with 100 μl of PBS (control; *n* = 4) or with 10 μg/ml of tobramycin (*n* = 8) for 24 h. Viability was assessed on control and tobramycin-treated tissue (*n* = 1) using MTT assay kit (MatTek Corp.). Cytotoxicity was measured by quantifying LDH activity in the underlying medium collected 24 h after treatment with PBS or tobramycin by using CytoTox96 nonradioactive cytotoxicity assay kit (Promega Corp.).

### Supporting Information

Figure S1 Verification of Stable Transfection of Retrocyclin Constructs Analysis of the genomic DNA and RNA of transfected HL60 cells confirms the stable transfection and transcription of rescued retrocyclin constructs, respectively. (A) PCR on genomic DNA template from transfected HL60 cells shows a 215-bp fragment representing retrocyclin cDNA construct and a 890-bp fragment of native retrocyclin gene in the genomic DNA of A1A3 clones but not in the VC cells.
(B, C) Correction of the premature termination codon of retrocyclin cDNA introduces an additional HpyCH4V restriction site in the middle of an 87-bp cDNA fragment. RNA isolated from HL60 cells (control, R1R3 clones 1 and 2, and A1A3 clones 1 and 2) was used to make cDNA. Retrocyclin constructs were amplified by PCR using the cDNA as template and digested using HpyCH4V restriction enzyme. Electrophoresis of the digested PCR products shows the expected 87-bp fragment in control cells (B) and the expected absence of 87-bp fragment in R1R3 and A1A3 clones (C). All the products were also verified by DNA sequencing.
(1.08 MB TIF)

Table S1 Primers Used for Verification of Retrocyclin Constructs (31 KB DOC)

## Abbreviations

A1A3: HL60 cells containing retrocyclin constructs A1 and A3
FITC: fluorescein isothiocyanate
HPLC: high-performance liquid chromatography
LDH: lactate dehydrogenase
PBMC: peripheral blood mononuclear cell
PVDF: polyvinylidene fluoride
R1R3: HL60 cells containing retrocyclin constructs R1 and R3
RP-HPLC: reverse-phase HPLC
SEM: standard error of the mean
VC: vector control
