# Supplementary figures and images for: Reawakening Retrocyclins: Ancestral Human Defensins Active Against HIV-1
Source: PLoS Biol. 2009 Apr 28;7(4):e1000095. doi: 10.1371/journal.pbio.1000095 (PMC2672613; doi:10.1371/journal.pbio.1000095)

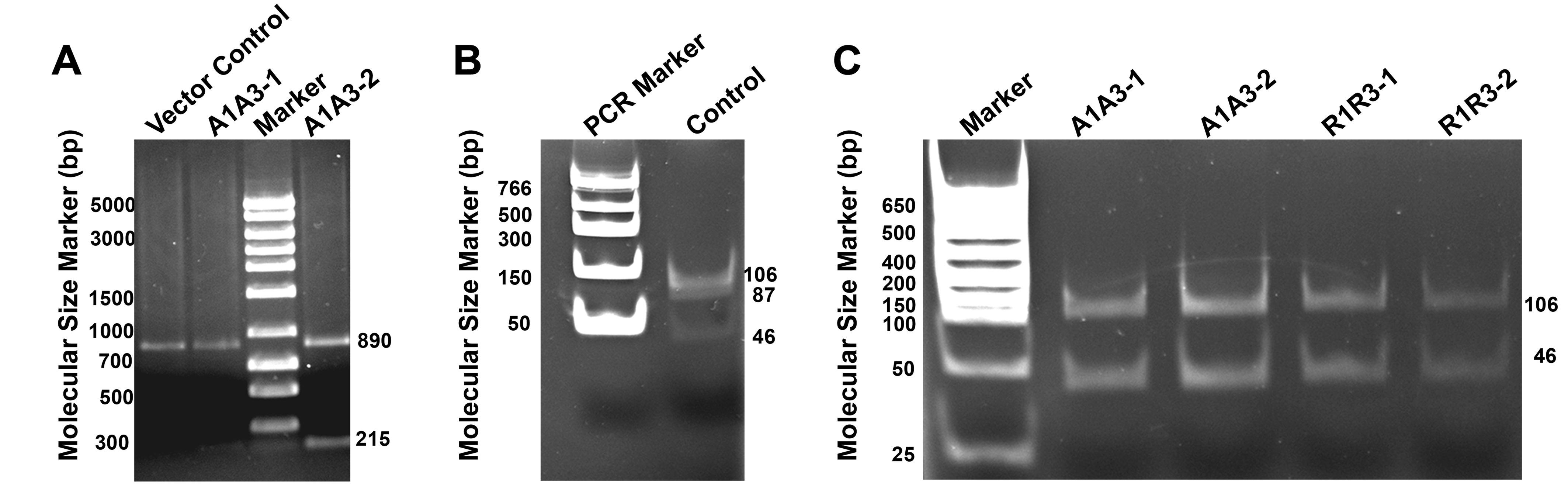

Supplement: Figure S1 — Analysis of the genomic DNA and RNA of transfected HL60 cells confirms the stable transfection and transcription of rescued retrocyclin constructs, respectively. (A) PCR on genomic DNA template from transfected HL60 cells shows a 215-bp fragment representing retrocyclin cDNA construct and a 890-bp fragment of native retrocyclin gene in the genomic DNA of A1A3 clones but not in the VC cells. (B, C) Correction of the premature termination codon of retrocyclin cDNA introduces an additional HpyCH4V restriction site in the middle of an 87-bp cDNA fragment. RNA isolated from HL60 cells (control, R1R3 clones 1 and 2, and A1A3 clones 1 and 2) was used to make cDNA. Retrocyclin constructs were amplified by PCR using the cDNA as template and digested using HpyCH4V restriction enzyme. Electrophoresis of the digested PCR products shows the expected 87-bp fragment in control cells (B) and the expected absence of 87-bp fragment in R1R3 and A1A3 clones (C). All the products were also verified by DNA sequencing. (1.08 MB TIF) [file pbio.1000095.sg001.tif]
