# Supplementary material for: Reawakening Retrocyclins: Ancestral Human Defensins Active Against HIV-1
Source: PLoS Biol. 2009 Apr 28;7(4):e1000095. doi: 10.1371/journal.pbio.1000095 (PMC2672613; doi:10.1371/journal.pbio.1000095)
Supplement: Table S1 — (31 KB DOC) [file pbio.1000095.st001.doc]

**Table S1:** Primers used for verification of retrocyclin constructs.

| **Primer**  **Name** | **Sequence** | **Accession Number** | **Primer Location** | **Template** |
| --- | --- | --- | --- | --- |
| DEFT_Fwd | TCCTCACTGCCATGCTTCT | AF526271.1 | 29-47 | Genomic DNA |
|  |  | AF355799 | 92-110 | cDNA |
| DEFT_Rev | TTATAACAAACGGCAAATTCCT | AF526271.1 | 897-918 | Genomic DNA |
|  |  | AF355799 | 285-306 | cDNA |
